# Remote Monitoring for Seizures During Therapeutic Hypothermia in Neonates With Hypoxic-Ischemic Encephalopathy

**DOI:** 10.1001/jamanetworkopen.2023.43429

**Published:** 2023-11-15

**Authors:** Gabriel Fernando Todeschi Variane, Alex Dahlen, Rafaela Fabri Rodrigues Pietrobom, Daniela Pereira Rodrigues, Maurício Magalhães, Marcelo Jenné Mimica, Nathalie Salles Llaguno, Danieli Mayumi Kimura Leandro, Paula Natale Girotto, Leticia Brito Sampaio, Krisa Page Van Meurs

**Affiliations:** 1 Division of Neonatology, Department of Pediatrics, Irmandade da Santa Casa de Misericórdia de São Paulo, São Paulo, Brazil; 2 Protecting Brains and Saving Futures Organization, Clinical Research Department, São Paulo, Brazil; 3 Quantitative Sciences Unit, Stanford University School of Medicine, Palo Alto, California; 4 Faculdade de Ciências Médicas da Santa Casa de São Paulo, São Paulo, Brazil; 5 Pediatric Nursing Department, Escola Paulista de Enfermagem, Universidade Federal de São Paulo, São Paulo, Brazil; 6 Division of Neurosurgery, Associação Paulista para o Desenvolvimento da Medicina, Hospital de Transplantes Euryclides de Jesus Zerbini, São Paulo, São Paulo, Brazil; 7 Division of Pediatric Neurology, Faculdade de Medicina Hospital das Clínicas, Instituto da Criança, Universidade de São Paulo, São Paulo, Brazil; 8 Division of Neonatal and Developmental Medicine, Stanford University School of Medicine and Lucile Packard Children’s Hospital Stanford, Palo Alto, California

## Abstract

**Importance:**

Neonates with hypoxic-ischemic encephalopathy (HIE) undergoing therapeutic hypothermia (TH) frequently experience seizures, which are associated with adverse outcomes. Efforts to rapidly identify seizures and reduce seizure burden may positively change neurologic and neurodevelopmental outcomes.

**Objective:**

To describe the onset, treatment, and evolution of seizures in a large cohort of newborns with HIE during TH assisted by a telehealth model and remote neuromonitoring approach.

**Design, Setting, and Participants:**

This was a prospective, observational, multicenter cohort study performed between July 2017 and December 2021 in 32 hospitals in Brazil. Participants were newborns with HIE meeting eligibility criteria and receiving TH. Data were analyzed from November 2022 to April 2023.

**Exposure:**

Infants with HIE receiving TH were remotely monitored with 3-channel amplitude-integrated electroencephalography (aEEG) including raw tracing and video imaging, and bedside clinicians received assistance from trained neonatologists and neurologists.

**Main Outcomes and Measures:**

Data on modified Sarnat examination, presence, timing and seizure type, aEEG background activity, sleep-wake cycling, and antiepileptic drugs used were collected. Descriptive statistical analysis was used with independent *t* test, χ^2^, Mann-Whitney test, and post hoc analyses applied for associations.

**Results:**

A total of 872 cooled newborns were enrolled; the median (IQR) gestational age was 39 (38-40) weeks, 518 (59.4%) were male, and 59 (6.8%) were classified as having mild encephalopathy by modified Sarnat examination, 504 (57.8%) as moderate, and 180 (20.6%) as severe. Electrographic seizures were identified in 296 newborns (33.9%), being only electrographic in 213 (71.9%) and clinical followed by electroclinical uncoupling in 50 (16.9%). Early abnormal background activity had a significant association with seizures. Infants with flat trace had the highest rate of seizures (58 infants [68.2%]) and the greatest association with the incidence of seizures (odds ratio [OR], 12.90; 95% CI, 7.57-22.22) compared with continuous normal voltage. The absence of sleep-wake cycling was also associated with a higher occurrence of seizures (OR, 2.22; 95% CI, 1.67-2.96). Seizure onset was most frequent between 6 and 24 hours of life (181 infants [61.1%]); however, seizure occurred in 34 infants (11.5%) during rewarming. A single antiepileptic drug controlled seizures in 192 infants (64.9%). The first line antiepileptic drug was phenobarbital in 294 (99.3%).

**Conclusions and Relevance:**

In this cohort study of newborns with HIE treated with TH, electrographic seizure activity occurred in 296 infants (33.9%) and was predominantly electrographic. Seizure control was obtained with a single antiepileptic drug in 192 infants (64.9%). These findings suggest neonatal neurocritical care can be delivered at remote limited resource hospitals due to innovations in technology and telehealth.

## Key Points

**Question:**

In a low- or middle-income country, what are the amplitude integrated electroencephalography characteristics in newborns with hypoxic-ischemic encephalopathy (HIE) undergoing therapeutic hypothermia?

**Findings:**

In this multicenter cohort study of 872 infants, seizures were identified in 33.9% of infants, and 71.9% of seizures were electrographic only. Seizure onset was most frequent in the first 24 hours (74.6%); however, 11.5% had onset during rewarming.

**Meaning:**

This large cohort study described brain monitoring characteristics in newborns with HIE, suggesting the feasibility and importance of a telehealth model and remote neuromonitoring approach in a low- or middle-income country.

## Teaser

This cohort study evaluates electroencephalography characteristics in newborns with hypoxic-ischemic encephalopathy (HIE) undergoing therapeutic hypothermia.

## Introduction

Perinatal asphyxia accounts for 23% of neonatal deaths worldwide, and it is also responsible for a substantial burden of long-term brain injury.^1,2,3,4,5^ It is estimated that hypoxic-ischemic encephalopathy (HIE) occurs in 1 to 3 cases per 1000 live births in high-income countries (HIC). In low- and middle-income countries (LMIC), these rates range from 5 to 26 cases per 1000 live births.^6,7,8^ In newborns with severe HIE, there is a high risk of death or disability, including cerebral palsy and mental retardation. Among babies with moderate HIE, sequelae include motor deficits, memory impairment, visual dysfunction, hyperactivity, and learning differences.^9,10,11,12^ Therapeutic hypothermia (TH) has been investigated in infants with moderate or severe HIE. In HIC, TH was shown to be a safe and beneficial intervention, significantly improving the combined outcome of mortality or major neurodevelopmental disability in patients aged 18 to 22 months and 6 to 7 years.^13,14,15^

Seizures occur in approximately 3.5 newborns per 1000 live births, and 40% to 60% of seizures in term newborn infants are caused by perinatal asphyxia, whereas in LMIC, the reported incidence of seizures is even higher.^16,17,18^ High seizure burden has been associated with worse neurologic outcomes and subsequent epilepsy.^19^ Recent investigations have demonstrated that more than 80% of seizures have no clinical correlate and early treatment decreases seizure burden.^20,21^ Despite this knowledge, many newborns do not undergo continuous electroencephalography (EEG) or amplitude-integrated EEG (aEEG) and thus are either not appropriately treated for seizures or are subject to receiving unnecessary antiepileptic medications.^21,22,23^

Continuous video–EEG (cEEG) is the criterion standard tool for neonatal seizure detection.^24^ However, significant barriers exist to its implementation since it requires experienced neurophysiologist interpretation, skilled technologists, and may not be readily available in all centers, especially in LMIC. The use of aEEG has increased in neonatal intensive care units (NICUs) as a simplified tool for continuous brain monitoring.^22,23^ It allows the assessment of background activity and sleep-wake cycling (SWC) as well as seizure identification in real time at the bedside. Brain monitoring guides appropriate therapeutic interventions such as the use of antiepileptic drugs (AEDs); furthermore, it provides useful information on estimation of outcomes in the setting of HIE.^25,26^ aEEG accompanied by raw EEG channels and video imaging (video aEEG/EEG) improves the accuracy of seizure detection with a sensitivity up to 85% and specificity of 90%; however, accuracy rates are highly dependent on user experience, and telehealth may play a role in providing the necessary expertise in aEEG/EEG monitoring in remote locations.^25,27,28,29^ Telehealth, including neuromonitoring and neurocritical care consultation, is the logical evolution of health care in the digital age, shortening distances, expanding access to and reach of specific methodologies, and lowering structural costs.^30,31,32^

The objective was to describe the video aEEG/EEG characteristics, including onset, treatment, evolution of seizures, background activity, and SWC in newborns with HIE during the cooling and rewarming periods assisted by a remote telemonitoring approach in an LMIC. This study also evaluated the possible association between seizures and additional aEEG/EEG abnormalities in this population.

## Methods

This multicenter, prospective, observational cohort study was conducted at 32 hospitals in Brazil, including 11 public, 14 private, and 7 public/private hospitals, during the period from July 2017 to December 2021. The study followed the Strengthening the Reporting of Observational Studies in Epidemiology (STROBE) reporting guidelines. It was approved by the institutional review boards at all the participating hospitals, and written parental consent was obtained. Protecting Brains and Saving Futures, a private company, provided remote neuromonitoring using an advanced telemedicine model consisting of longitudinal training, implementation of protocols, methodologies, real–time video aEEG/EEG monitoring (Neuro-Spectrum 4, [Neurosoft]), and neonatal and neurology consultations to deliver specialized neonatal neurocritical care around the clock.

All inborn infants requiring advanced resuscitation, with an acute perinatal event and a cord blood gas with pH less than 7.15, or a base deficit of 10 mmol/L or more were monitored with video aEEG/EEG starting during the first 6 hours of life. A clinical protocol for TH based on the National Institute for Child Health and Human Development (NICHD) whole-body hypothermia trial was developed together with a seizure management guideline (eAppendix in Supplement 1). Infants less than 35 weeks’ gestational age with birth weight under 1800 g or moribund condition were excluded.

All infants who met NICHD whole-body hypothermia trial eligibility indicating moderate or severe encephalopathy or who had seizure activity were treated with TH immediately. Infants with mild encephalopathy on the modified Sarnat examination and abnormal aEEG background activity with minimum amplitude below 5 μV and/or electrographic seizures were also cooled. Demographic and clinical data on newborns receiving TH were collected and analyzed.

All inborn infants treated with TH for HIE were monitored with 3-channel (C3-P3, C4-P4, and P3-P4) video aEEG/EEG^33,34,35^ reviewed by experienced readers located in a remote monitoring center. eFigure 1 in Supplement 1 shows the display available to the bedside clinicians and remote readers. Early background activity before 6 hours of life was described as continuous normal voltage (CNV), discontinuous normal voltage (DNV), continuous low voltage (CLV), burst-suppression (BS), or flat trace (FT), and SWC were classified according to Hellström-Westas.^36^ Electrographic seizure activity was defined as an abrupt increase in minimum and maximum amplitudes of aEEG associated with an evolving, stereotyped, and rhythmic wave pattern, with repeating forms such as spikes or sharp waves in raw EEG lasting at least 10 seconds.^37^ Single seizures were defined as 1 electrographic seizure per 30-minute epoch; repetitive seizures were defined as more than 1 electrographic seizure per 30-minute epoch but less than 1 electrographic seizure over a 10-minute period; status epilepticus was defined as continuous seizure activity for at least 30 minutes or recurring seizures for more than 50% of the recording time ranging from 1 to 3 hours.^38^ Seizure onset after birth was divided into 6 epochs: 0 to 6, 6 to 12, 12 to 24, 24 to 48, 48 to 72, and 72 to 96 hours of life. Time to normal trace (TTNT) was defined as the time in hours the infant took to have CNV background activity after birth.^39^

### Statistical Analysis

Demographic and clinical data were collected and correlated with video aEEG/EEG findings. Statistical analysis was performed with SPSS version 22.0 (IBM). Descriptive statistical analysis was used, and nonparametric variables were presented as median and IQR. The independent *t* test, χ^2^, Mann-Whitney test, and post hoc analyses were applied to assess the association between seizures and abnormal early background activity and absence of SWC. Tests were 2-sided with a significance threshold of .05. Data were analyzed from November 2022 to April 2023.

## Results

### Baseline Characteristics

Between July 2017 and December 2021, 1701 infants born in 32 hospitals in Brazil who required delivery room resuscitation or had a perinatal event were assessed for eligibility for cooling and 872 newborns received TH for HIE (eFigure 2 in Supplement 1). The number of participating hospitals and infants per year increased progressively, ranging from 7 hospitals and 48 infants in 2017 to 32 hospitals and 266 infants in 2021. The median (IQR) gestational age at birth was 39 (38-40) weeks, and 518 (59.4%) were male. Baseline characteristics are presented in Table 1, comparing infants with and without electrographic seizures. There were no differences in sex, mode of delivery, or gestational age. Significant differences were seen in Apgar score at 1, 5, and 10 minutes, as well as in pH and base deficit. According to the modified Sarnat examination,^40^ the majority of newborns had moderate HIE (504 patients [57.8%]). The Sarnat examination was not available for 129 infants (14.8%). In the group of infants initially classified with mild HIE (59 patients), 32 had electrographic seizures qualifying them for TH, while 12 presented with a continuous low voltage background pattern, defined as a minimum amplitude below 5 μV, leading to the decision to treat with TH. The remaining 15 met the laboratory criteria but had mild encephalopathy without electrographic abnormalities (deviated from eligibility in cooling protocol).

**Table 1.** Baseline Characteristics

Variable	Participants, No. (%)		*P* value	SD^a^
	HIE with seizures (n = 296)	HIE without seizures (n = 576)		
Sex	296	568		
Female	122 (41.2)	224 (39.4)	.11	−0.02
Male	174 (58.8)	344 (60.6)		0.02
Delivery	292	569		
Vaginal	128 (43.8)	265 (46.6)	.69	0.06
Cesarean	164 (56.2)	304 (53.4)		−0.06
Birth weight, median (IQR), g	3130 (2800-3435)	3113 (2800-3459)	.82	0.003
Gestational age, median (IQR), wk	39 (38-40)	39 (38-40)	.79	0.03
APGAR score at 1 min, median (IQR)	2 (1-3)	2 (1-4)	<.0001	−0.21
APGAR score at 5 min, median (IQR)	5 (3-6)	5 (4-7)	<.0001	−0.29
APGAR score at 10 min, median (IQR)	6 (4-7)	7 (5-8)	.001	−0.28
pH	143	312		
≤7.00	59 (41.2)	92 (29.6)	.006	−0.28
7.01 to 7.15	33 (23.1)	105 (33.6)		0.21
>7.15	51 (35.7)	115 (36.8)		0.07
Base deficit, mmol/L	133	303		
≥16	72 (54.1)	123 (40.6)	.005	−0.29
10 to 15.9	40 (30.1)	141 (46.5)		−0.32
<10	21 (15.8)	39 (12.9)		−0.03
Modified Sarnat examination	248	495		
Mild	32 (12.9)	27 (5.5)	<.0001	−0.28
Moderate	127 (51.2)	377 (76.2)		0.55
Severe	89 (35.9)	91 (18.4)		−0.42

Abbreviation: HIE, hypoxic-ischemic encephalopathy.

^a^ Standardized differences (SDs), also known as Cohen *d*, are a measure of effect size that can be interpreted as follows: SD = 0.2 corresponds to a small difference; 0.5, a medium difference; 0.8, a large difference; <0.2, a trivial difference.

### Brain Monitoring Findings

Video aEEG/EEG was initiated shortly after NICU admission and following confirmation of eligibility for TH, before 6 hours of life. The median (IQR) length of monitoring was 110 (92-136) hours in the newborns with seizures and 98 hours (49-111) for those without seizures. Electrographic seizures were identified in 296 neonates (33.9%). Seizures were electrographic only in 213 (71.9%); 50 neonates (16.9%) had electroclinical seizures followed by electroclinical uncoupling after treatment with AEDs, and the remaining 33 (11.1%) were electroclinical seizures. Single seizure was found in 48 infants (16.2%) and repetitive seizures in 170 (57.4%), while status epilepticus was present in 26 (8.8%). Fifty-two of the 296 infants with seizures (17.6%) did not have video recordings available for analysis, thus classification could not be performed.

**Figure 1.**
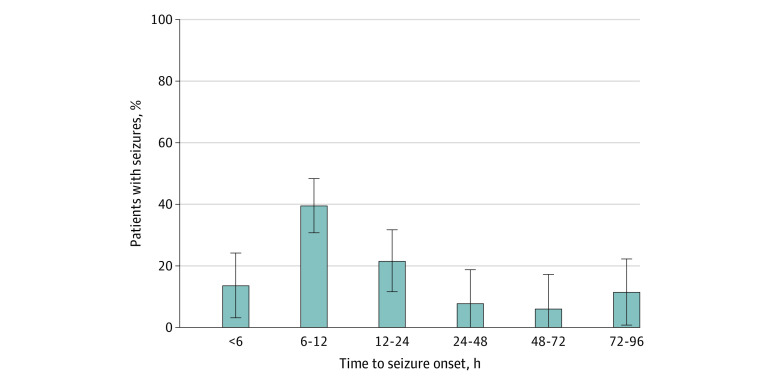
Time to Seizure Onset After Birth Seizure onset was more common between 6 to 24 hours of life, and a mild increase in seizure incidence is observed between 72 and 96 hours (rewarming period). Error bars indicate 95% CI.

**Figure 2.**
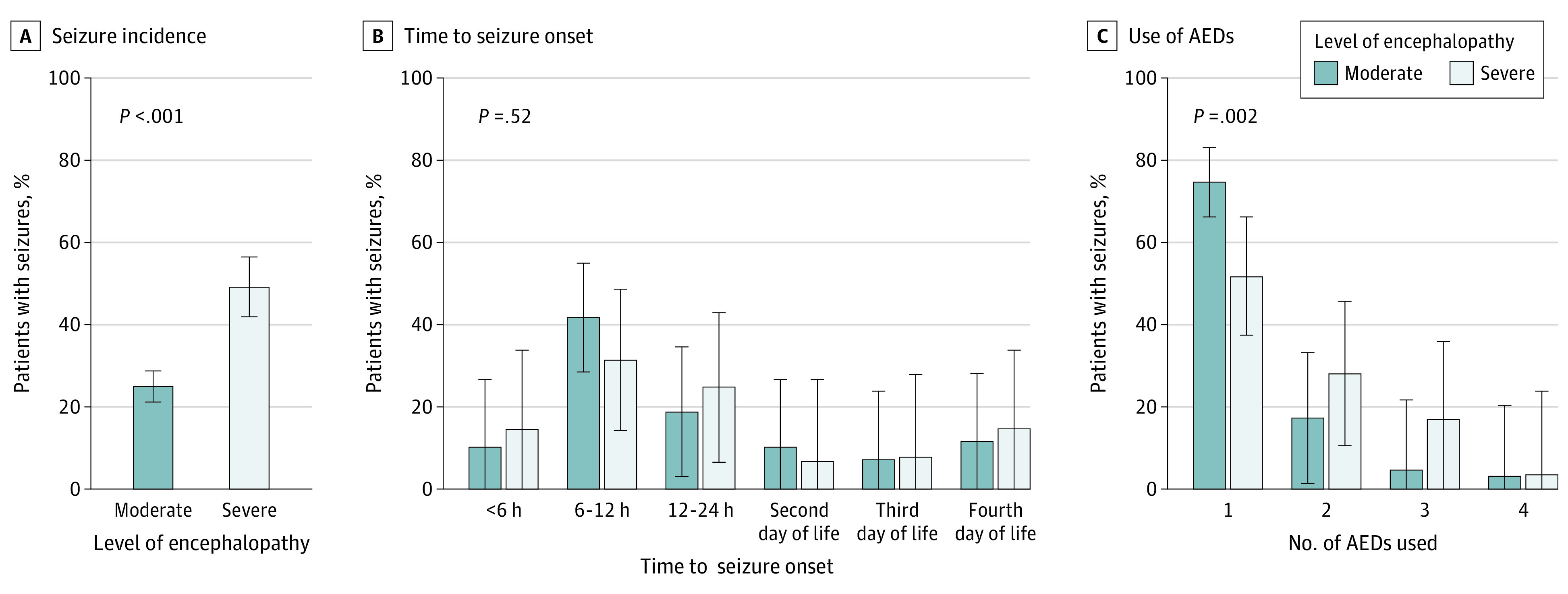
Seizure Incidence, Onset, and Use of Antiepileptic Drugs (AEDs) by Level of Encephalopathy Panel A, presence of severe encephalopathy was significantly associated with higher incidence of seizures (*P* < .001). Panel B, time to seizure onset was not significantly different by level of encephalopathy (*P* = .52). Panel C, number of AEDs used was significantly higher in infants with severe encephalopathy compared with those with moderate encephalopathy (*P* = .002). Error bars indicate 95% CI.

Seizure onset was most frequent between 6 and 12 hours of life (117 patients [39.5%]); 40 seizures (13.5%) occurred before 6 hours of life, and 181 seizures (61.1%) occurred between 6 and 24 hours of life, as shown in Figure 1. Seizure onset occurred during rewarming in 34 cases (11.5%). A single AED achieved seizure control in 192 infants (64.9%) while 69 (23.3%) required 2, 28 (9.5%) required 3, and 7 (2.4%) received 4 or more AEDs. Seventy infants (41.2%) with repetitive seizures and 20 (76.9%) with status epilepticus required 2 or more AEDs. Across all centers, the first line AED of choice was phenobarbital in 294 infants (99.3%), and the second line AED was either phenytoin in 87 (83.7%) or midazolam in 13 (12.5%). Seizure incidence, timing of onset, and use of AEDs by level of encephalopathy are shown in Figure 2.

Early background activity was evaluated as shown in Table 2, and 373 infants (42.8%) presented with CNV, 177 (20.3%) with DNV, 184 (21.1%) with CLV, 45 (5.2%) with BS, and 85 (9.7%) with FT. Background activity was not classified in 8 (0.9%) because they presented with status epilepticus. We found that most types of early abnormal background activity had a positive association with seizures. Infants with FT had the highest rate of seizures (58 infants [68.2%]) and the greatest association with the incidence of seizures (OR, 12.90; 95% CI, 7.57-22.22) compared with CNV. Infants with CNV had the lowest rate of seizures (53 infants [14.2%]).

**Table 2.** Correlation Between Early Background Activity, Sleep-Wake Cycling Findings, and Seizures

Characteristic	Participants, No. (%)			Odds ratio (95% CI)
	Overall (N = 872)	HIE with seizures (n = 296)	HIE without seizures (n = 576)	
Early background activity^a^				
Continuous normal voltage	373 (42.8)	53 (17.9)	320 (55.5)	1 [Reference]
Discontinuous normal voltage	177 (20.3)	72 (24.3)	105 (18.2)	4.14 (2.73-6.27)
Continuous low voltage	184 (21.1)	86 (29.1)	98 (17.1)	5.30 (3.52-7.96)
Burst suppression	45 (5.2)	19 (6.4)	26 (4.5)	4.41 (2.29-8.50)
Flat trace	85 (9.7)	58 (19.6)	27 (4.7)	12.9 (7.57-22.22)
Status epilepticus^b^	8 (0.9)	8 (2.7)	0	NA
Sleep-wake cycling^c^				
Present	425 (48.7)	106 (35.8)	319 (55.4)	1 [Reference]
Absent	447 (51.3)	190 (64.2)	257 (44.6)	2.22 (1.67-2.96)

Abbreviation: HIE, hypoxic-ischemic encephalopathy; NA, not applicable.

^a^ To assess early background activity, the initial background activity shown in the first 6 hours of life was considered.

^b^ When status epilepticus was present, background activity was not classified.

^c^ The presence of sleep-wake cycling and seizures was assessed during the entire monitoring time.

SWC was absent during the first day of life in 815 infants (93.5%) and absent in 447 newborns (51.3%) during the entire neuromonitoring period. There was a significant association between the absence of SWC and occurrence of seizures (OR, 2.22; 95% CI, 1.67-2.96). Early background activity and SWC findings in infants with and without seizures are shown in Table 2.

**Figure 3.**
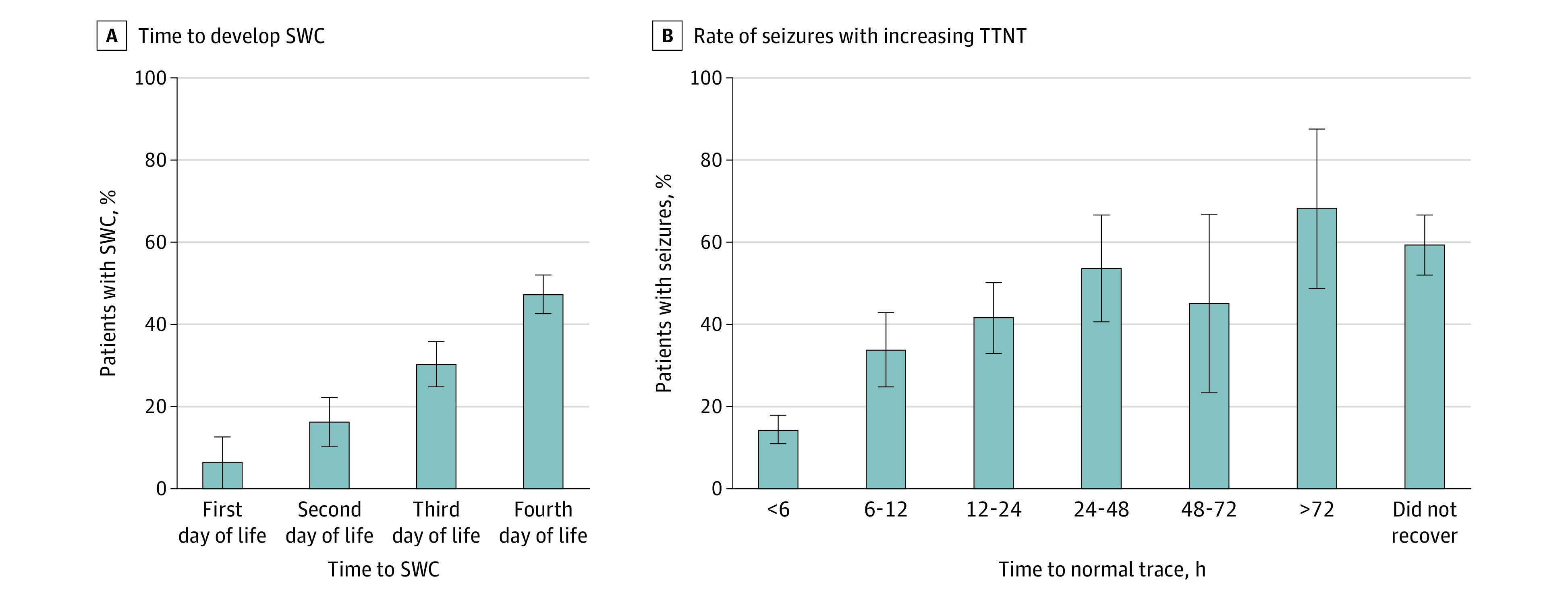
Time to Develop Sleep-Wake Cycling (SWC) and Rate of Seizures With Increasing Time to Normal Trace (TTNT) A, Presence of SWC increased with postnatal age but remained less than 50% by day 4 of life. B, Newborns with a shorter TTNT had a lower seizure incidence, while those with a longer TTNT had a higher seizure incidence. Error bars indicate 95% CI.

Seizures were present in 178 infants (26.3%) with TTNT within 72 hours of life and in 118 infants (60.2%) with TTNT after 72 hours of life. Time to develop SWC and the association between TTNT and seizures are shown in Figure 3.

## Discussion

This prospective, multicenter cohort study in Brazil describes the remote neuromonitoring findings using video aEEG/EEG in a cohort of 872 newborns treated with TH over a 4-year period. During the cooling and rewarming periods, 296 (33.9%) had electrographic seizures. Of the neonates with seizures, 263 (88.9%) were electrographic only or electroclinical followed by electrographic only. Seizure onset was most common during the first 24 hours of life; with 13.5% occurring before 6 hours of life and 61.1% between 6 and 24 hours of age. During the rewarming period, seizures occurred in 11.5%. A single AED was able to achieve seizure control in 64.9% infants. Phenobarbital was the first line AED used in 99.3% of cases. Seizures were more frequent in newborns who presented with abnormal early background activity or absence of SWC.

Previous publications report a seizure incidence during TH for HIE ranging from 22% to 65% of newborns, data compatible with the findings in this study (33.9%).^21,41^ In LMIC, the described seizure incidence is even higher. A recent multicenter randomized clinical trial (RCT) in India, Sri Lanka, and Bangladesh reported an incidence of seizures, diagnosed by clinical evaluation only, of 90% in cooled infants at a median (IQR) age onset of 2 (1.0-4.0) hours of life.^16^ Our findings differed from this published trial, with lower incidence and later onset of seizures. This may be explained by possible differences in the study populations, timing of hypoxic-ischemic insult, and use of aEEG/EEG for seizure identification. In the meantime, our findings on timing are similar to studies by Shah et al^42^ and Wusthoff et al.^41^ Shah et al^42^ assessed a cohort of 85 infants monitored with aEEG/EEG, which reported a seizure incidence of 52%. Wusthoff et al^41^ evaluated a cohort of 26 infants also monitored with aEEG/EEG with a seizure incidence of 65%, in which 19.2% commenced within the first 6 hours after birth, 27% at 6 to 12 hours, 23% between 12 and 18 hours, and 2% at 18 to 24 hours. These findings emphasize the importance of early brain monitoring in this population.

In our study, the majority of seizures were electrographic only (71.9%) similar to the existing literature.^21,43^ While performing continuous video–EEG, Murray et al^21^ described the recognition of clinical seizures by experienced neonatal clinicians. The authors reported that of 526 seizures identified with video-EEG, only 48 (9%) were correctly identified by clinical staff.

Several studies have demonstrated that phenobarbital is still the AED most used as the first line choice of treatment.^44,45^ Our study corroborates these findings and found that second-line drugs most used were phenytoin and midazolam (an intravenous levetiracetam preparation was not available in Brazil). Levetiracetam has been studied in neonates with the potential benefit of fewer adverse effects, however, pharmacokinetic, toxicity, and dosing information remain limited, and evidence shows lower efficacy when compared with phenobarbital as a first-line drug.^46^ The efficacy and safety of levetiracetam compared with phenobarbital as a first-line seizure treatment was recently reported in the NEOLEV2 multicenter RCT.^47^ In that study, phenobarbital controlled seizures in 80% of patients, compared with 28% in those receiving levetiracetam. This is similar to the seizure control achieved with phenobarbital in our study.

Abnormal initial background activity was associated with a higher risk for seizures. Macdonald-Laurs et al^48^ studied a cohort of 266 neonates with high risk or suspected seizures. They reported an association between the presence of seizures during cEEG monitoring and abnormalities in the first hour of monitoring. Previous studies^28,39^ have described that an abnormal aEEG tracing is associated with abnormal neurodevelopmental outcomes and a TTNT greater than 48 hours of life was a predictor of poor outcome. A systematic review by Del Rio et al^49^ included 17 studies and found that the maximum predictive reliability was at 72 hours of life in cooled infants with a posttest probability of 95.7% (95% CI, 84.4%-98.5%).

Our study reported that 42% of infants cooled had early normal background activity. Despite having early normal background activity, only 10.5% had SWC (eFigure 3 in Supplement 1) in the first day of life, and 14.2% developed seizures during the monitoring period. Several publications reported the presence of normal early aEEG background activity in infants with moderate or severe encephalopathy. Shankaran et al^14^ reported that 15 of 45 infants with an aEEG recording before 6 hours of life had a normal aEEG despite moderate or severe encephalopathy. In the same study, 12 out of 71 infants with moderate encephalopathy presented with CNV background activity before 9 hours of life. To increase the specificity of patient selection, the CoolCap trial^50^ had a 3-step protocol consisting of clinical evidence of exposure to perinatal hypoxia-ischemia, an abnormal neurological examination, and an abnormal aEEG. The requirement for an abnormal aEEG during the screening process excluded 98 infants from randomization. Previous studies showed that a normal aEEG is associated with better neurological outcomes in infants with HIE;^49^ however, there are still questions regarding the negative predictive value of a normal early aEEG. In a retrospective analysis Sarkar et al^51^ reported that 13 of 24 infants with HIE with an early normal aEEG had abnormal cranial magnetic resonance imaging. Although a normal aEEG may suggest a better prognosis for newborns with HIE, it should not exclude a baby otherwise eligible for TH according to blood gas and Sarnat examination.

To the best of our knowledge, this study describes seizure incidence and management in the largest cohort of infants with HIE monitored with video aEEG/EEG. Brain monitoring was started in the first 6 hours of life, and it was continued until the end of the rewarming period using remote monitoring by experienced neonatologists and neurologists. A telehealth model ensured consistent protocols, longitudinal training of NICU staff, real time consultations with the remote team, and aEEG/EEG monitoring by experienced readers. Expert interpretation and review of neuromonitoring is often not available outside of large, urban NICUs in HICs or LMICs. The use of remote neuromonitoring approach may allow more accurate seizure identification, appropriate use of AEDs, and earlier treatment of seizures. Fitzgerald et al^52^ showed that establishing a remote cEEG monitoring program for neonates is feasible, effective in detecting seizures, and enhances the quality of care delivered to neonates.

### Limitations

This study had limitations. In this observational study, we did not evaluate in-hospital or long-term outcomes. In addition, the use of aEEG/EEG instead of full montage video–EEG is also a limitation. Full montage video–EEG is the criterion standard method for seizure detection and aEEG/EEG may miss short, low-amplitude, and focal seizures.^5^ However, with experienced readers, aEEG/EEG has a reasonable accuracy for seizure detection with an estimated 85% sensitivity and 90% specificity.^53^ An important question is whether EEG monitoring is associated with improved neurological outcomes in this population. There is consensus regarding the importance of treatment and prevention of status epilepticus.^24^ Previous RCTs showed that the monitoring and treatment of electrographic seizures resulted in a significant reduction of seizure burden. Increased seizure burden was significantly associated with higher brain injury scores assessed by magnetic resonance imaging and lower performance in neurodevelopmental evaluation at 18 to 24 months.^17,54^ On the other hand, a recent RCT conducted by Hunt et al^55^ reported that treating electrographic seizures did not significantly reduce seizure burden and rate of death or disability at 2 years in a heterogeneous group of neonates; however, mean time of randomization of the intervention group was 26.8 hours of life, and it is possible that injury could have already occurred.

## Conclusions

HIE is a prevalent condition, especially in LMICs, and seizures are common in this population. Brain monitoring with aEEG/EEG or cEEG has a fundamental role in the care of these infants, as aggressive management of seizures with the goal of reducing seizure burden is thought to reduce brain injury and neurodevelopmental disability. Advances in information technology and telehealth enable the delivery of neonatal neurocritical care to remote centers with limited resources, thus achieving a higher level of care. Future studies should focus on the impact of this approach on neurological and neurodevelopmental outcomes.

## Notes

Supplement 1. **eAppendix.** Protecting Brains and Saving Futures Institutional Protocols **eFigure 1. **Display Available to the Bedside Clinicians and Remote Readers Showing 3-Channel Video aEEG/EEG During TH **eFigure 2. **STROBE Diagram **eFigure 3. **Presence of SWC by Early Background Activity

Supplement 2.
Data Sharing Statement
