# Supplementary material for: Remote Monitoring for Seizures During Therapeutic Hypothermia in Neonates With Hypoxic-Ischemic Encephalopathy
Source: JAMA Netw Open. 2023 Nov 15;6(11):e2343429. doi: 10.1001/jamanetworkopen.2023.43429 (PMC10652158; doi:10.1001/jamanetworkopen.2023.43429)
Supplement: Supplement 1. — eAppendix. Protecting Brains and Saving Futures Institutional Protocols eFigure 1. Display Available to the Bedside Clinicians and Remote Readers Showing 3-Channel Video aEEG/EEG During TH eFigure 2. STROBE Diagram eFigure 3. Presence of SWC by Early Background Activity [file jamanetwopen-e2343429-s001.pdf]

Supplemental Online Content

Variane GFT, Dahlen A, Rodrigues Pietrobom RF, et al. Remote monitoring for seizures during therapeutic hypothermia in neonates with hypoxic-ischemic encephalopathy. *JAMA Netw Open*. 2023;6(11):e2343429. doi:e2343429

eAppendix. Protecting Brains And Saving Futures Institutional Protocols

eFigure 1. Display Available to the Bedside Clinicians and Remote Readers Showing 3-Channel Video aEEG/EEG During TH

eFigure 2. STROBE Diagram

eFigure 3. Presence of SWC by Early Background Activity

This supplemental material has been provided by the authors to give readers additional information about their work.

eAppendix. Protecting Brains And Saving Futures Institutional Protocols

A. Therapeutic hypothermia protocol for newborns with hypoxic-ischemic encephalopathy

All newborns with a diagnosis of perinatal asphyxia or encephalopathy should be evaluated for eligibility to receive body cooling following the steps listed below. If the patient meets criteria for cooling, it should be started as soon as possible and within the first 6 hours after birth.

The assessment for Therapeutic Hypothermia includes cord gas or baby blood gas analysis in the first hour of life, assessment of clinical encephalopathy using the Modified Sarnat Scale, and, if available, the use of cerebral monitoring with amplitude-integrated electroencephalography.

Step 1: Identifying patients eligible for Therapeutic Hypothermia evaluation:

Newborns with gestational age greater than or equal to 35 weeks and birth weight greater than or equal to 1,800 grams, without major congenital malformations with the following additional criteria:

- Need for neonatal resuscitation in the delivery room and
- Suspected asphyxia with 5 minute APGAR score less than or equal to 7 and/or presence of an acute perinatal event.

Exclusion criteria:

- Gestational age below 35 weeks.
- Birth weight less than 1800 g.
- Severe congenital malformations and/or condition incompatible with life.

Step 2: Stabilization and transfer to the NICU:

- After stabilization in the Delivery Room, collect arterial or venous cord blood gases and/or blood gases from the newborn within the first hour of life;
- Resuscitation and transport to the Neonatal ICU according to the guidelines of the Brazilian Society of Pediatrics;
- Maintain thermal control in the Delivery Room and admission to the Neonatal ICU, maintaining normothermia until the level of encephalopathy has been determined. Avoid hyperthermia.

Step 3: Identifying infants with a potential hypoxic-ischemic insult:

- Cord blood gases or baby blood gases in the first hour of life with a pH less than or equal to 7.0 and/or BE less than or equal to -16

© 2023 Variane GFT et al. *JAMA Network Open.*

OR

- Baby blood gas in the first hour of life is not available (external transfer) OR if arterial blood gas analysis in the first hour of life shows a pH between 7.01 and 7.15 and/or BE between -10 and -15.9, evaluate the factors below:

• Presence of an acute perinatal event associated with either:

• Apgar score less than or equal to 5 in the 10th minute of life and/or;

• Need for ventilatory support for more than 10 minutes after birth.

Step 4: Assessment of level of encephalopathy:

This assessment should be performed using the Modified Sarnat Scale shown below and video aEEG/EEG, if available.

© 2023 Variane GFT et al. *JAMA Network Open.*

Category	Normal	Mild HIE	Moderate HIE	Severe HIE
**1) Level of Consciousness**	Alert, responsive	Hyperalert, responds to minimal stimuli	Lethargic	Stupor or Coma
**2) Spontaneous activity**	Spontaneous	Spontaneous or decreased	Decreased	No activity
**3) Posture**	Normal	Mild distal flexion (wrist and fingers)	Strong distal flexion or full extension	Decerebrate
**4) Tone**	In normal flexed position	In normal flexed position	Mild Hypotonia (focal or general) or hypertonia	Flaccid or rigid
**5) Primitive reflexes**				
**Suck**	Strong	Weak	Weak	Absent
**Moro**	Complete	Normal or incomplete	Incomplete	Absent
**6) Autonomic System**				
**Pupils**	Reactive	Dilated	Constricted	Non-reactive
**Heart rate**	100 to 160 bpm	Tachycardia	Bradycardia	Variable
**Breathing**	Regular	Tachypnea	Periodic breathing	Apnea

If one of the following are present, initiate TH:

• Three or more criteria of moderate or severe HIE from the Modified Sarnat Scale and/or;

• Clinical seizures and/or;

• Video aEEG/EEG with abnormal cerebral background activity (defined as minimum amplitude below 5 μV) and/or presence of seizure.

Step 5:

If patient meets the criteria shown above and there are no exclusion criteria, cooling should be started when the eligibility criteria are met and before 6 hours of life. Maintain rectal or esophageal temperature

© 2023 Variane GFT et al. *JAMA Network Open.*

between 33°C to 34°C for 72 hours. Rewarming should be started after 72 hours of cooling, increasing rectal or esophageal temperature at a rate of 0.2-0.5°C per hour.

Indication for therapeutic hypothermia in the neonatal ICU

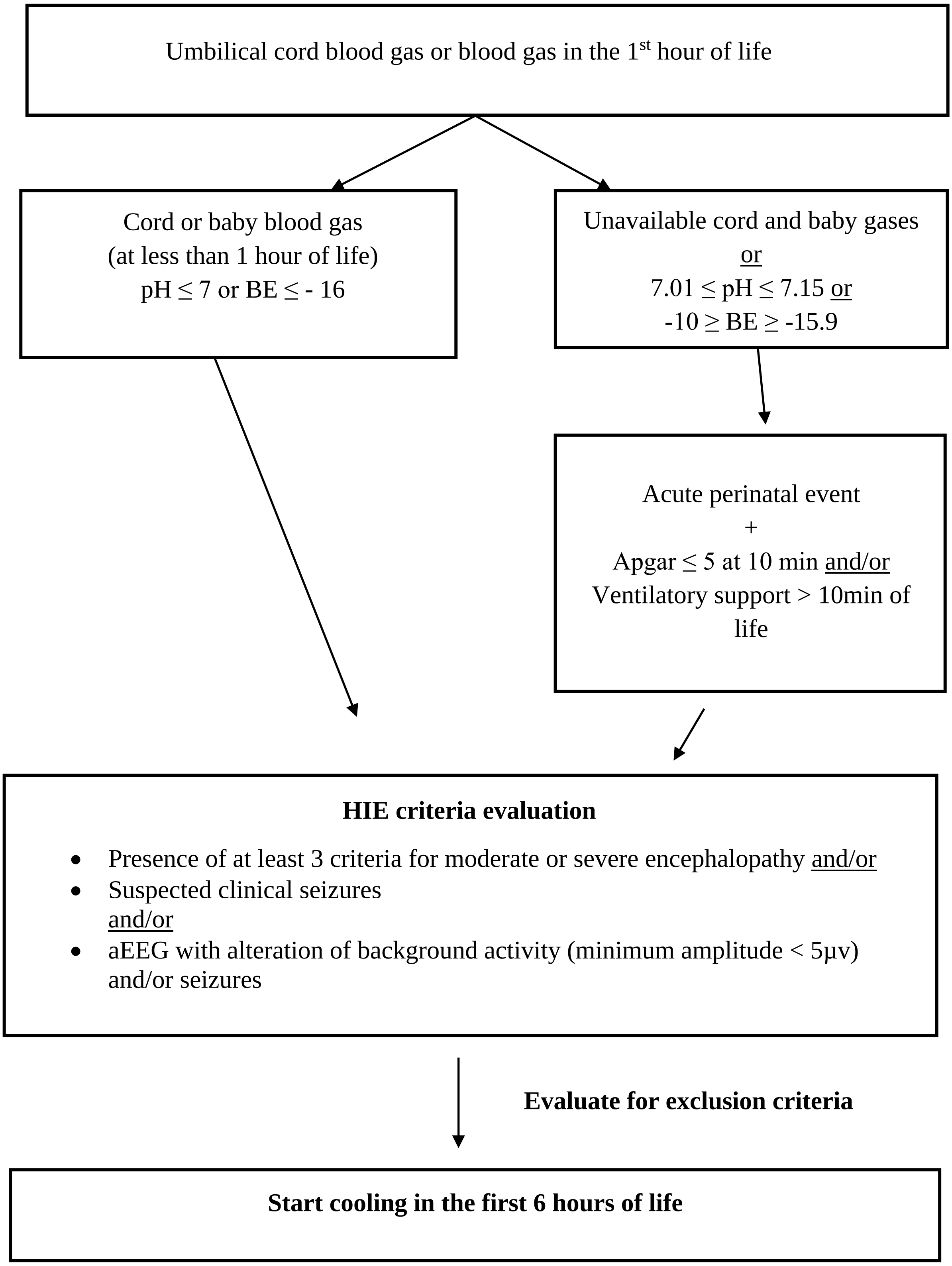

© 2023 Variane GFT et al. *JAMA Network Open.*

B. Neonatal seizure management during therapeutic hypothermia

Seizures are common during therapeutic hypothermia. Follow the algorithm below for seizure management.

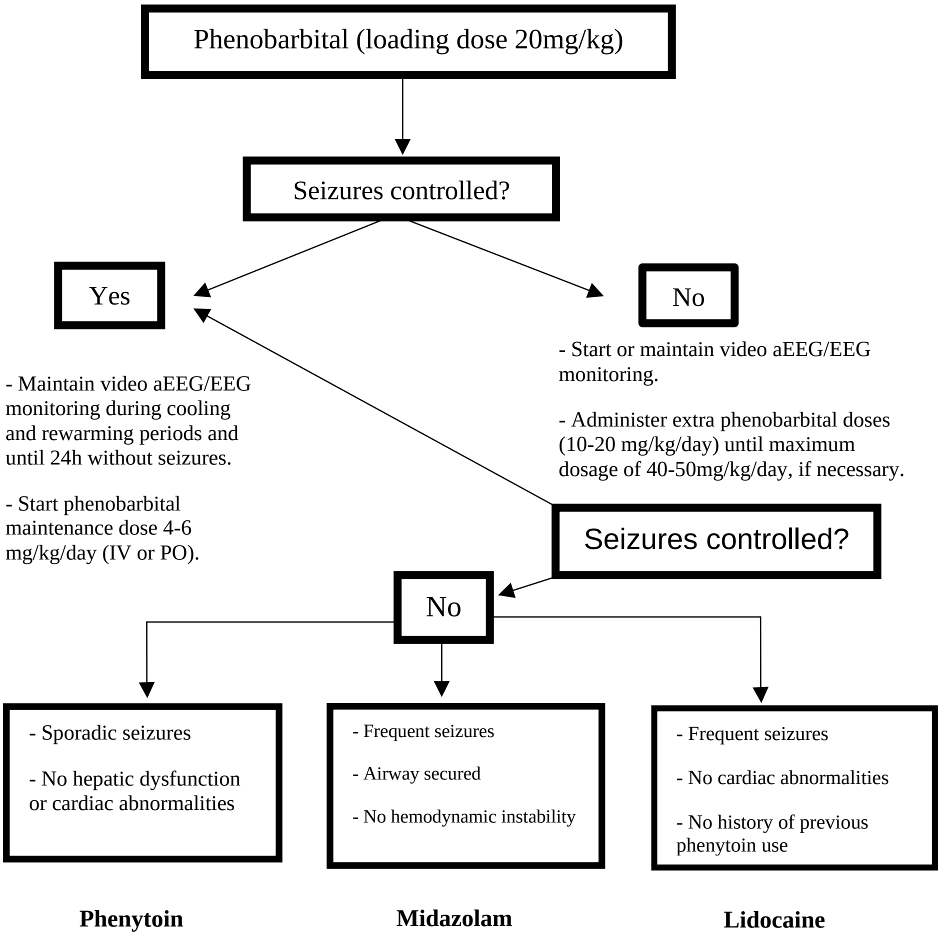

Figure 1: Neonatal seizure management algorithm.

- **Phenobarbital:** The initial recommended dose of phenobarbital is 20mg/kg. In patients who do not respond to an initial bolus, additional doses may be given up to a maximum of 40-50 mg/kg/day before starting a second antiepileptic drug.
- **Phenytoin:** Loading dose of 20mg/kg followed by a maintenance dose of 5-8 mg/kg/day IV divided in 2 to 3 doses.
- **Midazolam:** Can be started with an initial bolus dose followed by continuous infusion titrated until the therapeutic effect is achieved. It can be used for consecutive days. Weaning should be started after 24 hours without seizures. For continuous intravenous administration a secure airway is

recommended. As the main adverse effect is hypotension, it is not recommended for patients with hemodynamic instability.

- **Lidocaine:** May be effective for status epilepticus treatment. It is contraindicated in newborns who have recently received phenytoin or who have congenital heart disease, due to the risk of cardiac arrhythmia. To avoid toxicity, lidocaine infusion should not be continued for more than 30 hours. The dose of lidocaine should be adjusted to birth weight and exposure or not to therapeutic hypothermia treatment (see Table 1).

This protocol did not include levetiracetam as a second line option to treat neonatal seizures because in Brazil there is no IV formulation available.

Table 1: Suggested lidocaine infusion protocol

**Weight**	**Bolus (over 10min)**	**Initial infusion**	**2nd Infusion**	**3rd infusion**	**Treatment duration**	**Dose Total (mg/kg)**
**Normothermia**						
< 2.5 kg	2 mg/kg	6 mg/kg/h for 4h	3 mg/kg/h for 12h	1.5 mg/kg/h for 12h	28h	80
≥ 2.5kg	2 mg/kg	7 mg/kg/h for 4h	3.5 mg/kg/h for 12h	1.75 mg/kg/h for 12h	28h	93
**Hypothermia**						
< 2.5 kg	2 mg/kg	6 mg/kg/h for 3.5h	3 mg/kg/h for 12h	1.5 mg/kg/h for 12h	27.5h	77
≥ 2.5kg	2 mg/kg	7 mg/kg/h for 3.5h	3.5 mg/kg/h for 12h	1.75 mg/kg/h for 12h	27.5h	89.5

Seizures refractory to antiepileptic drugs:

If seizures do not respond to antiepileptic drugs treatment and the etiology is uncertain, a therapeutic trial of pyridoxine should be considered, so that pyridoxine-dependent epilepsy is ruled out. A loading dose of 100mg IV, followed by a maintenance dose of 15-30mg/kg/day (maximum 200mg/day) divided into 2 doses for 3 days is recommended. Patients should receive cardiorespiratory monitoring during the administration of the initial doses.

Further etiological investigation in these cases is necessary, especially to exclude neonatal epilepsy and inborn errors of metabolism.

eFigure 1. Display Available to the Bedside Clinicians and Remote Readers Showing 3-Channel Video aEEG/EEG During TH

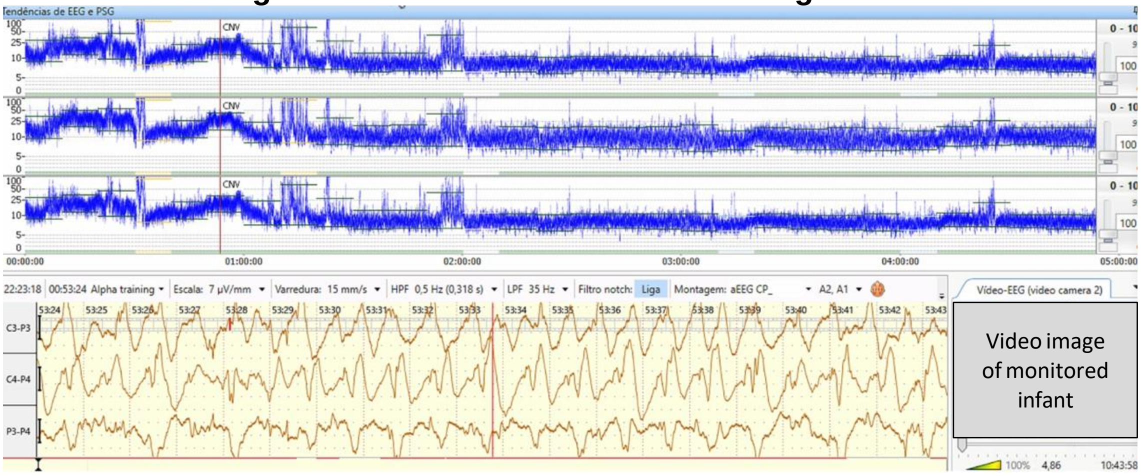

The first tracings (in blue) represent the 3 channel (C3-P3; C4-P4; P3-P4) aEEG, and 3 traces below (in brown) represent the raw EEG channels, along with the video image of the infant.

© 2023 Variane GFT et al. *JAMA Network Open.*

eFigure 2. STROBE Diagram

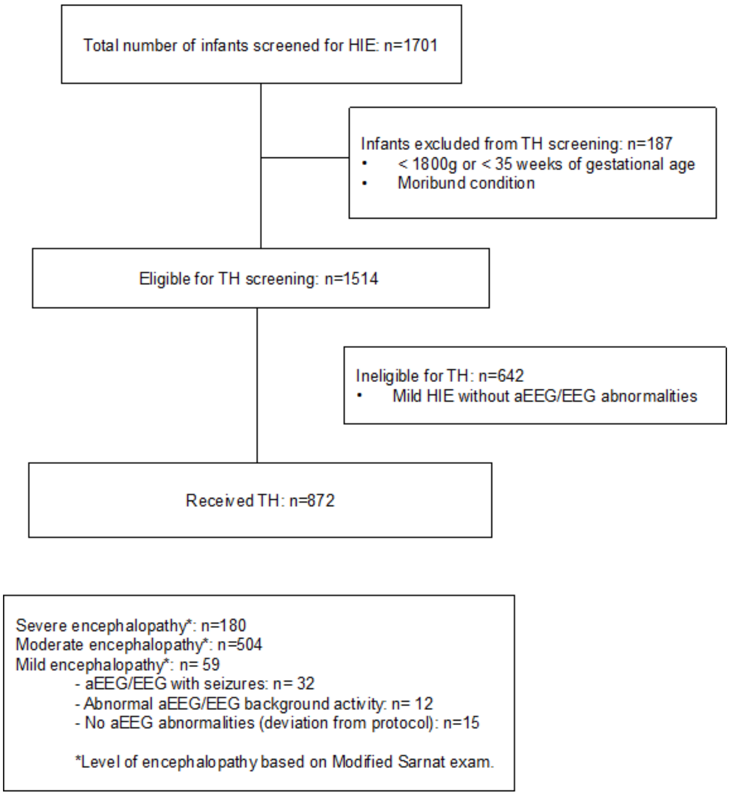

© 2023 Variane GFT et al. *JAMA Network Open.*

eFigure 3. Presence of SWC by Early Background Activity

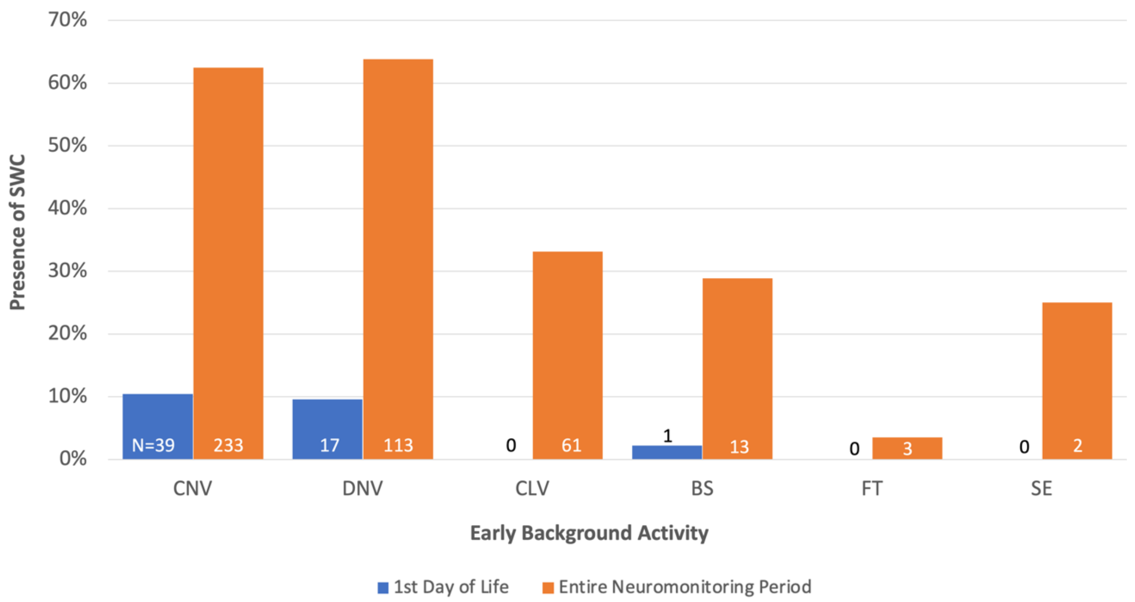

Early background activity was commonly CNV or DNV, however early SWC was infrequent among all infants included in this study.

© 2023 Variane GFT et al. *JAMA Network Open.*
