# Supplementary material for: Remote Monitoring for Seizures During Therapeutic Hypothermia in Neonates With Hypoxic-Ischemic Encephalopathy
Source: JAMA Netw Open. 2023 Nov 15;6(11):e2343429. doi: 10.1001/jamanetworkopen.2023.43429 (PMC10652158; doi:10.1001/jamanetworkopen.2023.43429)
Supplement: Supplement 2. — Data Sharing Statement [file jamanetwopen-e2343429-s002.pdf]

Data Sharing Statement

Variane. Remote Monitoring for Seizures During Therapeutic Hypothermia in Neonates With Hypoxic-Ischemic Encephalopathy. *JAMA Netw Open*. Published November 16, 2023. doi:10.1001/jamanetworkopen.2023.43429

Data
Data available: No

Additional Information
Explanation for why data not available: Unidentified study data may be shared upon request.
